# Prevalence of cancer risk factors among transgender and gender diverse individuals: a cross-sectional analysis using UK primary care data

**DOI:** 10.3399/BJGP.2023.0023

**Published:** 2023-06-27

**Authors:** Jalen Brown, Ruth M Pfeiffer, Duncan Shrewsbury, Stewart O’Callaghan, Alison M Berner, Shahinaz M Gadalla, Meredith S Shiels, Sarah S Jackson

**Affiliations:** Division of Cancer Epidemiology and Genetics, National Cancer Institute, Rockville, MD, US.; Division of Cancer Epidemiology and Genetics, National Cancer Institute, Rockville, MD, US.; Department of Medical Education, Brighton and Sussex Medical School, Brighton, UK.; Live Through This Charity, London, UK.; Barts Cancer Institute, Queen Mary University of London, London, UK; Gender Identity Clinic, Tavistock and Portman NHS Foundation Trust, London, UK.; Division of Cancer Epidemiology and Genetics, National Cancer Institute, Rockville, MD, US.; Division of Cancer Epidemiology and Genetics, National Cancer Institute, Rockville, MD, US.; Division of Cancer Epidemiology and Genetics, National Cancer Institute, Rockville, MD, US.

**Keywords:** cancer, health disparities, LGBTQ, morbidity, transgender persons

## Abstract

**Background:**

Transgender and gender diverse (TGD) individuals experience an incongruence between their assigned birth sex and gender identity. They may have a higher prevalence of health conditions associated with cancer risk than cisgender people.

**Aim:**

To examine the prevalence of several cancer risk factors among TGD individuals compared with cisgender individuals.

**Design and setting:**

A cross-sectional analysis was conducted using data from the UK’s Clinical Practice Research Datalink to identify TGD individuals between 1988–2020, matched to 20 cisgender men and 20 cisgender women on index date (date of diagnosis with gender incongruence), practice, and index age (age at index date). Assigned birth sex was determined from gender-affirming hormone use and procedures, and sex-specific diagnoses documented in the medical record.

**Method:**

The prevalence of each cancer risk factor was calculated and the prevalence ratio by gender identity was estimated using log binomial or Poisson regression models adjusted for age and year at study entry, and obesity where appropriate.

**Results:**

There were 3474 transfeminine (assigned male at birth) individuals, 3591 transmasculine (assigned female at birth) individuals, 131 747 cisgender men, and 131 827 cisgender women. Transmasculine people had the highest prevalence of obesity (27.5%) and ‘ever smoking’ (60.2%). Transfeminine people had the highest prevalence of dyslipidaemia (15.1%), diabetes (5.4%), hepatitis C infection (0.7%), hepatitis B infection (0.4%), and HIV infection (0.8%). These prevalence estimates remained elevated in the TGD populations compared with cisgender persons in the multivariable models.

**Conclusion:**

Multiple cancer risk factors are more prevalent among TGD individuals compared with cisgender individuals. Future research should examine how minority stress contributes to the increased prevalence of cancer risk factors in this population.

## INTRODUCTION

Transgender and gender diverse (TGD) individuals experience an incongruence between their assigned sex at birth and gender identity. Between 0.1% and 2.0% of the worldwide population identify as TGD.^1^ Several cancer risk factors, including obesity, alcohol use, exogenous hormone use, smoking, and viral infections are associated with multiple cancer types.^2^^,^^3^ The prevalence of these risk factors among TGD people has not been well characterised.

The minority stress framework^4^ posits that institutionalised stigma and social norms marginalise TGD individuals resulting in chronic stress.^5^ For example, 23% of transgender people in the US have stated they avoided seeking necessary medical care in the past year due to discrimination and stigma.^6^

Individuals reported experiencing harassment from clinicians or refusal of care because of their gender identity. These experiences may lead TGD persons to delay medical care or participate in harmful behaviours that can impact the prevention and treatment of conditions that predispose to cancer. Indeed, studies have shown associations between discrimination against TGD people in health care and increased tobacco use.^7^^,^^8^ Transgender individuals may be more likely to smoke and to have alcohol use disorders (for example, substance misuse and alcohol poisoning) than cisgender individuals.^9^^,^^10^ Additionally, TGD individuals present on average with more comorbidities than cisgender people and may be at a higher risk of most chronic conditions, including obesity and dyslipidaemia.^11^

The evidence regarding the effects of gender-affirming hormone therapy on long-term health is mixed.^12^ Gender-affirming hormone therapy can produce physiological and metabolic changes that require monitoring.^13^^–^15 Testosterone and oestrogen use has been linked with short-term changes in body mass index (BMI) and lean body mass. Unfavourable changes in lipid composition have been associated with testosterone and oestrogen use,^16^^,^^17^ particularly among transmasculine individuals.^18^

Much of the literature to date is cross-sectional or limited to TGD individuals on gender-affirming hormone therapy, which overlooks the social and environmental conditions affecting the TGD experience. Furthermore, an understanding of these morbidities in the context of cancer risk is necessary as this population ages. Given these gaps in the literature, this study focused on estimating the prevalence of key cancer risk factors in the TGD community compared with cisgender people.

**Table:** How this fits in

Transgender and gender diverse (TGD) individuals experience an incongruence between their assigned sex at birth and gender identity. Little research has been conducted on the prevalence of cancer risk factors among TGD individuals. In this analysis using primary care data it was found that factors such as smoking, alcohol use, obesity, dyslipidaemia, diabetes, and HIV and hepatitis infections are elevated among TGD persons, likely due to the increased stigma and discrimination this population faces. Awareness of the higher prevalence of these risk factors among TGD people can enable GPs to offer more opportunistic patient education and investigation.

## METHOD

A cross-sectional analysis of risk factors for cancer was conducted using the UK’s Clinical Practice Research Datalink (CPRD). CPRD is a longitudinal primary care database that includes patients across participating practices within the UK.^19^^,^^20^ Data was combined from CPRD GOLD (which includes patients from England, Wales, Scotland, and Northern Ireland) and CPRD Aurum (which includes patients from England only).^19^^,^^20^ CPRD is representative of the UK and comparable to the UK census in terms of age, sex, and ethnicity.^19^^,^^20^ There were 7151 TGD individuals diagnosed with gender incongruence (formerly gender identity disorder) from 1988 to 2020 aged ≥18 years using Read and SNOMED codes from GOLD and Aurum, respectively (see Supplementary Tables S1 and S2). The index date was defined as the first occurrence of a gender incongruence diagnosis among TGD individuals. Each TGD person was individually matched to 20 cisgender men and 20 cisgender women from the same medical practice on index age (age at gender incongruence diagnosis, ±1 year) and index year (year of gender incongruence diagnosis, ±1 year). The matched cisgender cohort consisted of 140 983 cisgender men and 141 060 cisgender women. Exclusion criteria were applied to exclude individuals:

- with a diagnosis of gender incongruence who were believed to be misclassified cisgender people, such as those taking finasteride for benign prostatic hypertrophy, or individuals taking menopausal hormone therapy after a hysterectomy or mastectomy;
- in CPRD Aurum who had been referred to LGBT services only and no other gender incongruence codes with no evidence of gender affirming hormone therapy or surgery (∼1.7% of TGD persons in Aurum);
- over the age of 90 years; and
- with variations of sex characteristics (formerly disorders of sex development; see Supplementary Tables S3 and S4).

The final analysis population consisted of 6603 TGD adults matched to 263 574 cisgender adults (see Supplementary Figure S1). The cancer risk factors of interest included smoking status (current, former, or never smoker), alcohol use (current, former, or never user), and obesity (BMI ≥30 kg/m^2^), obtained from the first documentation of the condition closest to the index date. Chronic conditions like HIV infection, hepatitis B infection, hepatitis C infection, dyslipidaemia, and diabetes were based on documentation of diagnosis codes or medications related to the diagnosis closest to the index date.

### Statistical analyses

The prevalence of each risk factor by gender identity was estimated using Poisson regression with sandwich estimator for factors with high prevalence (all except for viral infection outcomes) or log binomial regression to yield the prevalence ratio (PR) with a 95% confidence interval (CI).^21^^,^^22^ All models were adjusted for continuous index age and continuous index year. Models were further adjusted for dyslipidaemia and diabetes, and for obesity.

Sex assigned at birth was determined from the medical record based on a combination of gender-affirming hormone therapy and procedures, and sex-specific diagnosis terms (see Supplementary Tables S5–S7). Because the authors were unable to identify the sex assigned at birth for a total of 3725 TGD individuals, multiple imputation was performed for missing values in sex assigned at birth, in addition to missing values for BMI, alcohol use, and smoking status based on height, weight, index age, index year, and all cancer risk factors. Multiple imputation was performed using proc MI in SAS (version 9.4) to create five imputed datasets. PROC SURVEYFREQ and PROC MIANALYZE (SAS, version 9.4) was used to obtain pooled frequencies and proportions. In a sensitivity analysis, frequencies, proportions, and PRs were reported without imputation. The analysis where sex assigned at birth was imputed but individuals with missing smoking use, alcohol use, and/or BMI data were excluded are also reported. All counts less than five are suppressed per CPRD policy to protect privacy. All analyses were carried out in accordance with CPRD guidelines and regulations.

This work is a collaboration between authors who are researchers, community organisers, advocates for, and members of the TGD community. TGD insight was instrumental during the design and execution of this research. This analysis was reported in accordance with the Strengthening the Reporting of Observational Studies in Epidemiology guidelines.^23^

## RESULTS

The analysis included 6603 TGD individuals (see Supplementary Table S8) and, following imputation, 3258 TGD persons were categorised as transmasculine and 3345 persons as transfeminine (see Table 1). The matched cisgender cohort consisted of 131 747 cisgender men and 131 827 cisgender women. The mean age at index date for transmasculine people was 30.2 years and the mean age for transfeminine people was 35.6 years.

**Table 1.** Characteristics of transgender and gender diverse individuals and cisgender individuals in CPRD 1988–2020 using multiple imputation^a^

**Characteristic**	***n* (%)^b^**			
	**Transmasculine people (*n* = 3258)**	**Transfeminine people (*n* = 3345)**	**Cisgender men (*n* = 131 747)**	**Cisgender women (*n* = 131 827)**
**Age, mean (SE)**	30.2 (0.22)	35.6 (0.26)	32.9 (0.04)	32.8 (0.04)

**Body mass index**				
Underweight/normal	1600 (49.1)	1605 (47.9)	61 543 (46.7)	67 157 (51.0)
Overweight	773 (23.7)	1018 (30.4)	43 435 (33.0)	33 178 (25.2)
Obese	884 (27.5)	721 (21.6)	26 770 (20.3)	31 492 (23.9)

**Smoking Status**				
Never	1299 (39.9)	1216 (36.3)	55 138 (41.9)	60 650 (46.0)
Former	980 (30.1)	1001 (30.0)	33 083 (25.1)	37 156 (28.2)
Current	980 (30.1)	1128 (33.7)	43 527 (33.0)	34 021 (25.8)

**Alcohol use**				
Never	484 (14.9)	328 (9.8)	13967 (10.6)	17 522 (13.3)
Former	278 (8.5)	193 (5.8)	5484 (4.2)	7556 (5.7)
Current	2496 (76.6)	2824 (84.4)	112 296 (85.2)	106 750 (81.0)

**Dyslipidaemia**				
Yes	280 (8.6)	507 (15.1)	14 839 (11.3)	11 127 (8.4)
No	2978 (91.4)	2838 (84.8)	116 908 (88.7)	120 700 (91.6)

**Diabetes**				
Yes	150 (4.6)	179 (5.4)	5732 (4.4)	5359 (4.1)
No	3108 (95.4)	3166 (94.6)	126 015 (95.6)	126 468 (95.9)

**Hepatitis C infection**				
Yes	13 (0.4)	22 (0.7)	459 (0.3)	258 (0.2)
No	3245 (99.6)	3323 (99.3)	131 288 (99.7)	131 569 (99.8)

**Hepatitis B infection**				
Yes	9 (0.3)	15 (0.4)	372 (0.3)	313 (0.2)
No	3249 (99.7)	3330 (99.6)	131 375 (99.7)	131 514 (99.8)

**HIV infection**				
Yes	18 (0.5)	28 (0.8)	317 (0.2)	173 (0.1)
No	3240 (99.4)	3317 (99.2)	131 430 (99.8)	131 654 (99.9)

a *Numbers and percentages may not add up or round to the total due to the imputation procedure.*

b *Unless otherwise stated. CPRD = Clinical Practice Research Datalink.*

Transmasculine people had the highest prevalence of obesity (27.5%) but the lowest prevalence of current alcohol use (76.6%). Transfeminine individuals had the highest prevalence of current smoking (33.7%), dyslipidaemia (15.1%), and diabetes (5.4%). HIV infection was higher among transmasculine individuals (0.5%) and transfeminine individuals (0.8%), compared with cisgender men (0.2%) and cisgender women (0.1%) (Table 1).

In the multivariable models, obesity was elevated for transmasculine individuals compared with cisgender men (PR 1.39; 95% CI = 1.30 to 1.49) and cisgender women (PR 1.17; 95% CI = 1.09 to 1.26) (see Table 2). Transfeminine adults had a lower prevalence of obesity than cisgender women (PR 0.88; 95% CI = 0.80 to 0.95) but the same prevalence as cisgender men (PR 1.02; 95% CI = 0.93 to 1.11). Transmasculine adults had a higher prevalence of dyslipidaemia compared with cisgender women (PR 1.31; 95% CI = 1.15 to 1.48), but not compared with cisgender men (PR 0.94; 95% CI = 0.83 to 1.06). Transfeminine adults had elevated prevalence of dyslipidaemia compared with cisgender men (PR 1.12; 95% CI = 1.02 to 1.22) and cisgender women (PR 1.53; 95% CI = 1.40 to 1.68). Diabetes prevalence was elevated for transmasculine adults compared with cisgender men (PR 1.24; 95% CI = 1.04 to 1.47) and cisgender women (PR 1.29; 95% CI = 1.09 to 1.53). Also, transfeminine adults showed elevated diabetes prevalence compared with cisgender women (PR 1.24; 95% CI = 1.06 to 1.45), but not compared with cisgender men (PR 1.05; 95% CI = 0.90 to 1.23).

**Table 2.** Prevalence ratios of cancer risk factors for transgender and gender diverse individuals compared with cisgender individuals in the UK’s Clinical Practice Research Datalink (with imputation)

**Outcome**	**Transmasculine people versus cisgender men, PR (95% CI)**	**Transmasculine people versus cisgender women, PR (95% CI)**	**Transfeminine people versus cisgender men, PR (95% CI)**	**Transfeminine people versus cisgender women, PR (95% CI)**
Obesity (BMI ≥30 kg/m^2^)^a^	1.39 (1.30 to 1.49)	1.17 (1.09 to 1.26)	1.02 (0.93 to 1.11)	0.88 (0.80 to 0.95)
Current smoker^a^	1.00 (0.93 to 1.08)	1.23 (1.14 to 1.32)	1.05 (0.99 to 1.13)	1.30 (1.22 to 1.39)
Former smoker^a^	1.27 (1.18 to 1.35)	1.21 (1.13 to 1.30)	1.11 (1.04 to 1.18)	1.11 (1.04 to 1.19)
Current alcohol use^a^	0.95 (0.91 to 0.99)	0.98 (0.94 to 1.02)	1.00 (0.96 to 1.04)	1.04 (1.00 to 1.08)
Former alcohol use^a^	1.33 (1.17 to 1.51)	1.27 (1.12 to 1.44)	1.13 (0.97 to 1.32)	1.15 (0.99 to 1.35)
Dyslipidaemiaa	0.94 (0.83 to 1.06)	1.31 (1.15 to 1.48)	1.12 (1.02 to 1.22)	1.53 (1.40 to 1.68)
Diabetes^a^^,^^b^	1.24 (1.04 to 1.47)	1.29 (1.09 to 1.53)	1.05 (0.90 to 1.23)	1.24 (1.06 to 1.45)
HIV infection^c^	2.40 (1.43 to 4.02)	4.41 (2.60 to 7.45)	3.29 (2.20 to 4.91)	6.02 (3.98 to 9.12)
Hepatitis C infection^c^	1.27 (0.72 to 2.23)	2.21 (1.25 to 3.91)	1.71 (1.11 to 2.63)	3.10 (2.00 to 4.82)
Hepatitis B infection^c^	1.05 (0.53 to 2.08)	1.23 (0.62 to 2.44)	1.48 (0.87 to 2.50)	1.78 (1.05 to 3.01)

a *Prevalence ratios were estimated using multiple imputation to impute missing values for sex assigned at birth, BMI, smoking, and alcohol use with log-binomial regression adjusted for age, index year, and index practice.*

b *Models also adjusted for obesity.*

c *Prevalence ratios were estimated using multiple imputation to impute missing values for sex assigned at birth, BMI, smoking, and alcohol use with Poisson regression with a robust variance estimator adjusted for age, index year, and index practice.*

*BMI = body mass index. PR = prevalence ratio.*

Compared with cisgender women, current smoking was elevated for both transmasculine individuals (PR 1.23; 95% CI = 1.14 to 1.32) and transfeminine individuals (PR 1.30; 95% CI = 1.22 to 1.39). Compared with cisgender men, there was no difference in the current smoking prevalence for either transfeminine adults (PR 1.05; 95% CI = 0.99 to 1.13) or transmasculine adults (PR 1.00; 95% CI = 0.93 to 1.08) (Table 2).

For former smoking, transfeminine people showed elevated prevalence compared with cisgender men (PR 1.11; 95% CI = 1.04 to 1.18) and cisgender women (PR 1.11; 95% CI = 1.04 to 1.19). Transmasculine people were also more likely to be former smokers than cisgender men (PR 1.27; 95% CI = 1.18 to 1.35) and cisgender women (PR 1.21; 95% CI = 1.13 to 1.30) (Table 2).

Compared with cisgender men, transmasculine adults were less likely to be current alcohol users (PR 0.95; 95% CI = 0.91 to 0.99), but transfeminine adults were not (PR 1.00; 95% CI = 0.96 to 1.04). Transmasculine individuals (PR 0.98; 95% CI = 0.94 to 1.02) and transfeminine individuals (PR 1.04; 95% CI = 1.00 to 1.08) were just as likely to be current drinkers as cisgender women. Transmasculine individuals were more likely to be former drinkers than cisgender men (PR 1.33; 95% CI = 1.17 to 1.51) and cisgender women (PR 1.27; 95% CI = 1.12 to 1.44). Transfeminine individuals were just as likely to be former drinkers compared with cisgender men (PR 1.13; 95% CI = 0.97 to 1.32) and cisgender women (PR 1.15; 95% CI = 0.99 to 1.35) (Table 2).

Compared with cisgender men, transmasculine people (PR 2.40; 95% CI = 1.43 to 4.02) and transfeminine people (PR 3.29; 95% CI = 2.20 to 4.91) had an elevated prevalence of HIV infection. Compared with cisgender women, there was an increased prevalence of HIV infection among transmasculine people (PR 4.41; 95% CI = 2.60 to 7.45) and transfeminine people (PR 6.02; 95% CI = 3.98 to 9.12) (Table 2).

Among transmasculine people, hepatitis C infection was two times higher compared with cisgender women (PR 2.21; 95% CI = 1.25 to 3.91), but not elevated compared with cisgender men (PR 1.27; 95% CI = 0.72 to 2.23). The prevalence of hepatitis C infection was three times higher for transfeminine people compared with cisgender women (PR 3.10; 95% CI = 2.00 to 4.82), and almost two times higher compared with cisgender men (PR 1.71; 95% CI = 1.11 to 2.63). Hepatitis B prevalence for transmasculine individuals was not elevated compared with cisgender women (PR 1.23: 95% CI = 0.62 to 2.44) or compared with cisgender men (PR 1.05; 95% CI = 0.53 to 2.08). Transfeminine adults had an elevated prevalence of hepatitis B infection compared with cisgender women (PR 1.78; 95% CI = 1.05 to 3.01), but not compared with cisgender men (PR 1.48; 95% CI = 0.87 to 2.50) (Table 2).

In sensitivity analyses, where the prevalence and prevalence ratios were calculated without imputing assigned birth sex (see Supplementary Table S9) and individuals with missing smoking, alcohol, and BMI information were removed (see Supplementary Table S10), the results did not materially differ.[Fig f1]

## DISCUSSION

### Summary

In this large analysis using primary care data, it was found that there is an increased prevalence of cancer risk factors among TGD individuals. Transmasculine individuals showed an elevated prevalence of obesity, smoking, dyslipidaemia, and hepatitis C infection compared with cisgender women and an elevated prevalence of obesity, current alcohol use, diabetes, and HIV infection compared with cisgender men. Transfeminine individuals showed elevated prevalence of smoking, dyslipidaemia, diabetes, and hepatitis B, hepatitis C, and HIV infections compared with cisgender women, but a decreased prevalence of obesity.

Transfeminine people also showed elevated dyslipidaemia, hepatitis C infection, and HIV infection compared with cisgender men. These findings suggest that minority stress due to stigma and discrimination, in addition to factors like hormone use, may increase comorbidity risk.

**Figure 1. f1:**
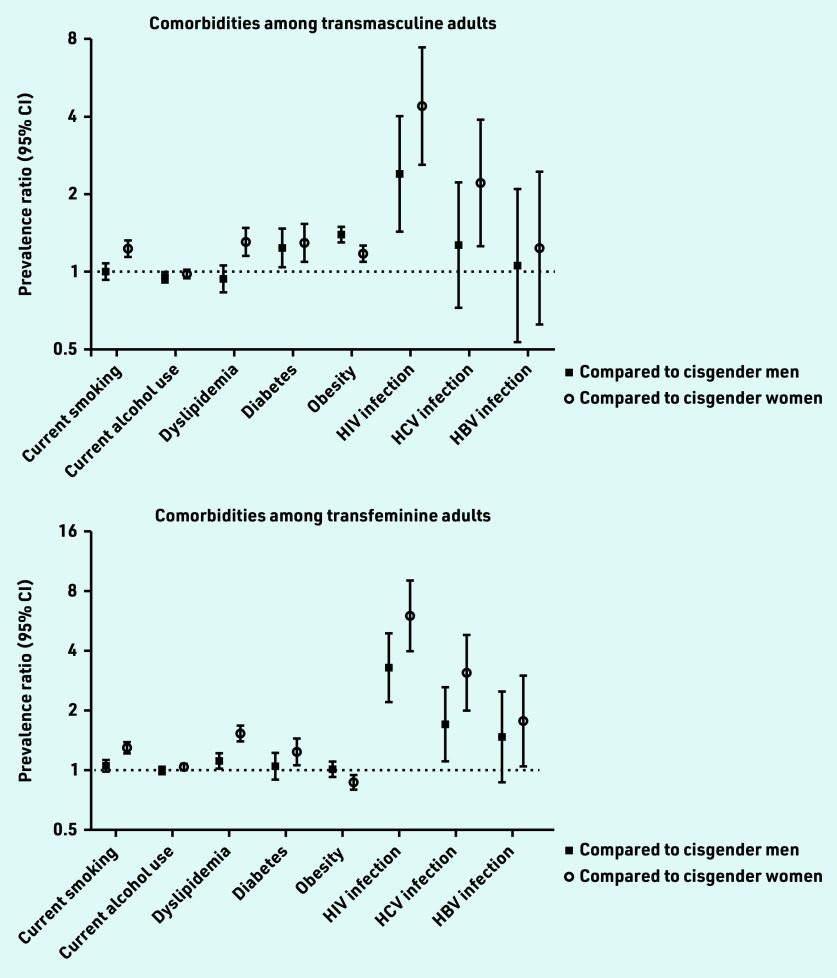
*Prevalence ratios for common comorbidities among transmasculine and transfeminine adults compared with cisgender men and cisgender women.*

### Strengths and limitations

A strength of this study is the inclusion of TGD individuals using diagnosis codes for gender incongruence and not exclusive to those receiving gender-affirming care such that the results may be more generalisable to the wider TGD community engaged in health care. However, as TGD people are less likely to engage with the medical system than cisgender people,^6^^–^8^,^^24^ these results may not be generalisable to all TGD adults in the UK. Diagnosis codes and medications were used to define many of the risk factors of interest, which are likely to be more accurate than self-report.

A limitation of this study is that TGD identity was inferred based on gender incongruence codes and assumed sex assigned at birth through electronic medical record information, as opposed to self-reported gender identity as is the gold standard.^25^ Relying on diagnosis codes for gender incongruence may miss people who have not disclosed to their providers or who do not seek medical transition.

Furthermore, the prevalence ratios may be inflated if clinicians were more likely to ascertain health history from TGD persons than cisgender persons due to ascertainment bias. The prevalence of some conditions may have been underestimated by using diagnosis codes and medications rather than laboratory values.^26^ Lastly, the authors were unable to adjust for important confounders, including socioeconomic status, race/ethnicity, income level, physical activity, and immigration status due to the lack of documentation of these factors in CPRD.

In this article, the terms transmasculine (assigned female at birth) and transfeminine (assigned male at birth) have been used to best represent the potential gender diversity captured by the study’s inclusion criteria. Terminology used by clinical and TGD populations can vary, with the most widely accepted terms often being introduced and upheld by the latter.^27^ This, along with the study's intention to balance inclusivity and specificity, forms the rationale for the choice of terminology. Due to the nature of the CPRD data it is difficult to reliably identify and report on non-binary identities. The authors recognise this limitation and acknowledge that this should be addressed in future research.

### Comparison with existing literature

Current literature suggests that transmasculine individuals experience an increase in body mass related to hormone therapy.^16^^,^^17^^,^^28^ However, the increased prevalence of obesity among this population compared with cisgender individuals may also involve social and environmental factors such as living in poverty or reduced physical activity.^29^^,^^30^ Changes to lipid profiles shortly after initiating gender-affirming hormone therapy have been documented in transfeminine and transmasculine adults and adolescents.^18^^,^^31^ Specifically unfavourable changes, such as an increase in total cholesterol, triglycerides, and low-density lipoprotein cholesterol levels along with a decrease in high-density lipoprotein cholesterol levels was observed for transmasculine individuals.^18^ However, these changes may not necessarily equate to poor cardiovascular outcomes.

A systematic review of TSG individuals on gender-affirming hormone therapy, showed very few deaths due to stroke and myocardial infarction, especially among transgender men.^32^ The present study found higher diabetes prevalence among TGD individuals compared with cisgender individuals; however, studies from Europe and the US have found no increase in diabetes prevalence among TGD populations.^33^^–^35

Previous studies have shown higher tobacco use among TGD individuals compared with cisgender individuals.^9^^,^^11^^,^^36^ In this study, the cohort includes patients seeking primary health care, many of whom are seeking gender-affirming care that may require cessation of smoking.^37^ Regarding alcohol use, a decreased prevalence of self-reported alcohol use was observed among Veterans Health Administration TSG patients in the US; however, the same study also found an increase in alcohol use disorder diagnoses relative to cisgender patients.^10^ Likewise, Hughto *et al*^11^ showed that TGD individuals had a higher prevalence of alcohol use disorder diagnoses compared with cisgender people. It is possible that the findings from the present study that former alcohol use is elevated among transmasculine individuals reflects abstinence following an alcohol use disorder diagnosis.

This study also found that the prevalence of HIV infection was two to six times higher among TGD individuals than cisgender individuals. Additionally, hepatitis C infection was two to three times higher among TGD individuals compared with cisgender women. Increased prevalence, incidence, and diagnosis of HIV infection may be due to engaging in condomless sexual intercourse, possibly in the context of survival sex work and/or injection drug use.^38^^–^40 The population included in the present study had a low overall prevalence of HIV compared with national estimates of 0.46–4.78 per 1000 TSG persons,^41^ likely due to the study population being engaged with primary care and potentially more aware of prevention measures. However, these data suggest that the HIV and hepatitis C epidemics for TGD persons, particularly transfeminine individuals, are still ongoing and that targeted interventions are needed to reduce the number of newly acquired infections each year.

### Implications for research and practice

Chronic health conditions may be increased among TGD patients for a number of reasons, including, but not limited to, minority stress due to societal discrimination and stigma. Chronic stress from institutionalised stigma and social norms results in TGD individuals’ rejection of healthcare needs as a priority, resulting in worse health outcomes.^24^^,^^42^^,^^43^ GPs should be aware of the increased risk of chronic conditions among TGD patients to provide proper prevention and treatment. For example, the increased prevalence of smoking and alcohol use among TGD patients in this study cohort suggests that harm reduction or cessation counselling in primary care settings may significantly benefit TGD patients.

Additionally, GPs should be aware that a significant amount of discrimination occurs in healthcare settings, with more than half of TGD people reporting avoiding going to a doctor when feeling unwell.^24^ A 2021 survey of almost 700 TSG individuals in the UK found that 70% experienced transphobia in medical settings and 14% reported being refused health care (of any kind) by a GP for being transgender.^24^ These instances were more common for non-binary and Black people and people of colour.^24^ Consequently, TGD individuals may delay addressing their healthcare needs in the face of this stigma and stress, resulting in worse health outcomes. GP practices may wish to undertake additional training for all staff to address discrimination.^44^^–^46 Awareness of delayed presentations by TGD people may enable GPs to offer more opportunistic patient education or investigation when patients do present.

This analysis of TGD individuals in primary care found an elevated prevalence of at least one risk factor for cancer, including viral infections, such as HIV, as well as diseases of metabolic origin, like obesity, diabetes, and dyslipidaemia. Reasons impacting the presence of these risk factors may include social and environmental determinants of health that remain underaddressed in this population. Further longitudinal research is required to elucidate the factors driving the increase of these morbidities and if these factors result in increases in diseases like cancer in this population.

## Data

The data that support the findings of this study are available from CPRD, but restrictions apply to the availability of these data, which were used under license for the current study, and so are not publicly available. Data are however available with permission of CPRD.
